# Changes in BMI prior to and during the COVID-19 pandemic among children: a retrospective cohort study in San Francisco, CA

**DOI:** 10.1186/s12889-024-20311-4

**Published:** 2024-10-25

**Authors:** Sarah L. Maxwell, Charles E. McCulloch, Alicia Fernandez, Amy L. Beck

**Affiliations:** 1 Department of Pediatrics, University of California, San Francisco, 550 16th Street, 4th Floor, San Francisco, California 94158 USA; 2 Department of Epidemiology and Biostatistics, University of California, San Francisco, San Francisco, California USA; 3 Department of Medicine, University of California, San Francisco, San Francisco, California USA

**Keywords:** Overweight/obesity, COVID-19 pandemic, School closures, Health disparities

## Abstract

**Background:**

The COVID-19 pandemic led to dramatic changes in the lives of children that impact cardiometabolic health. Cities and counties had varying policies with respect to school closure, recreational programs, and efforts to mitigate food insecurity and economic distress. Our objective was to evaluate changes in BMI-z score and prevalence of overweight/obesity prior to and during the pandemic among children in San Francisco, CA, where public schools were closed for 18-months.

**Methods:**

This was an electronic medical record-based retrospective cohort study. We included 15,401 children, 4–17 years of age at study onset. Our exposure was time into each of three time periods: (1) March 2018-February 2019; (2) March 2019-February 2020; (3) March 2020-August 2021 (the pandemic period of school closure). Generalized estimating equations (GEE) were used to assess changes in BMI-z score and overweight/obesity across the three time periods. We assessed for effect modification by age-category, insurance status, and race/ethnicity.

**Results:**

Mean BMI-z score increased by 0.06 per year in time period 2, the year prior to the pandemic (*p* < 0.001, 95% CI: 0.04, 0.09), and by 0.12 per year during time period 3, the first 18 months of the pandemic (*p* < 0.001, 95% CI 0.10, 0.13). The proportion of children with overweight/obesity increased by 1.4% points per year during time period 2 (*p* = 0.012, 95% CI: 0.03, 2.46) and by 4.9% points per year during the first 18 months of the pandemic (*p* < 0.001, 95% CI: 4.11, 5.67). The effect modification analysis demonstrated that the youngest age group, publicly insured children (versus privately insured), and Black, Latino, and Asian children (versus White children) experienced greater increases in BMI-z score during the pandemic (*p* < 0.01 for all comparisons). The youngest age group (*p* = 0.022) and publicly insured children (versus privately insured children) (*p* < 0.001) also experienced greater increases in the proportion of children with overweight/obesity during the pandemic.

**Conclusions:**

Among children in San Francisco, increases in BMI-z score and overweight/obesity were greater during the pandemic compared to prior changes, with the most pronounced increases among younger and publicly insured children. These findings support the need for more targeted and effective policies for addressing childhood overweight/obesity, especially among these high-risk populations.

## Background

Policies to reduce the spread of COVID-19, including closures of schools, playgrounds, and recreational programs, led to significant changes in children and adolescents’ daily routines, with more time spent at home and, for many, fewer opportunities to engage in physical activity and access healthy meals [1–6]. These policies may have had unintended impacts.

A number of studies have monitored the impacts of the pandemic on childhood obesity. Some of the early studies point to increases in the rate of weight gain among children, in the first year of the pandemic [1–5, 7–10], and among school aged children [1, 2, 7, 9]. These studies have also found differential impacts on weight and overweight/obesity among younger children [2, 7], by race/ethnicity [1, 3, 4, 9], and by insurance status [1, 3, 4]. Two more recent retrospective cohort studies have looked at the impacts over 18–30 months of the pandemic [10, 11] A study in Indiana found persistent increases in severe obesity for all children during 2020 and 2021, and for overweight and obesity among children ages 5–11 years [11]. A recent study in Philadelphia found significant increases in obesity rates early in the pandemic, followed by a significant decrease, and the trend returning to pre-pandemic levels by December 2022 [10]. Nearly all of these studies have focused on the experiences of children in specific geographic locations.

This geographic focus is important as the policy responses to the pandemic varied among state and local governments and across public school districts. San Francisco, CA, is a key locale for studying the impacts of COVID-19 pandemic on overweight/obesity in children as the public health measures were more restrictive and the school closures longer than in many other locations in the country. In San Francisco, public schools remained remote for most students through August 2021, resulting in a cumulative 18-months without in-person school [12]. San Francisco also experienced prolonged closures of recreational activities for children that provide opportunities for physical activity. Thus, San Francisco is an important area for evaluating the impacts of pandemic policies on BMI in children.

Our San Francisco based study aimed to evaluate changes in BMI-z scores and BMI category (overweight/obesity vs. no overweight/ obesity) prior to and during the pandemic among a racially and ethnically diverse group of children and adolescents 4–17 years of age receiving primary care at an academic medical center and a federally qualified health center in San Francisco, CA.

## Methods

### Study design and study subjects

This was a retrospective electronic medical record (EMR) based cohort study. Participants were children and adolescents who receive primary care at the University of California San Francisco Health primary care sites located in San Francisco and at San Francisco General Hospital (SFGH). Changes in population level BMI z-scores and BMI category were assessed across three time periods: (1) March 2018-February 2019 (pre-COVID-19, period 1), (2) March 2019-February 2020 (pre-COVID-19, period 2), and (3) March 2020-August 2021 (during the COVID-19 pandemic, period 3). The third time period, which was 18-months in duration, was chosen because this was the length of the school closures for most children attending public school in San Francisco. Children were included if they were 4–17 years of age at the start of the study and had at least one BMI measurement in the medical record prior to the COVID-19 pandemic (time period 1 or time period 2) and one BMI measurement during the COVID-19 pandemic (time period 3).We intentionally included all participants with one measurement in time period 1 or 2 and one measurement in time period 3, rather than limiting to those with measurements in each of the three time periods to avoid selection bias (as children who come more regularly to medical visits may be systematically different than those who do not). Our methodology allows for robust estimates even with missing data. No participants were excluded due to any demographic characteristics or co-morbid conditions. The study was approved by the Institutional Review Board (IRB) of the University of California, San Francisco (UCSF).

### Measures

The primary predictor was time into each study time period and the primary outcome was BMI-z score. A BMI-z score represents the BMI measurement’s distance from the mean BMI value for age and sex. BMI-z score is calculated as the difference between the BMI and the age- and sex-specific mean divided by the age-and sex-specific standard deviation [13]. Our secondary outcome was BMI category dichotomized into overweight/obesity vs. no overweight/obesity using age and sex adjusted BMI percentiles [13]. Both BMI z-score and BMI category were defined using CDC growth curves [13]. We abstracted baseline age-category, sex, race/ethnicity, and insurance status (private versus public) from the medical record. Baseline age was categorized into four groups: 4–6 year olds, 7–9 year olds, 10–12 year olds, and 13–17 year olds. Race and ethnicity were determined from the medical record. Within both health systems that contributed data to the study, patients and families are asked to self-report race/ethnicity. We categorized ethnicity as a binary variable: Latino or Not Latino. We then created the following race/ethnicity categorical variables: Latino; Black; White; and Asian. As there were small numbers of both American Indians/Alaskan Indians and Pacific Islanders/Native Hawaiians, our moderation analyses focused on the four most common racial/ethnic groups. Insurance status was determined from the documentation in the medical record at the time of most recent visit. Insurance was categorized as a binary variable: public or private.

### Statistical analyses

We used descriptive statistics to calculate baseline characteristics of the study population. Generalized estimating equations (GEE) were used to model population level change in BMI-z score and the proportion of overweight/obesity within each of the three time periods. For BMI-z score we used linear regression models. For overweight/obesity we used a generalized linear model with a binomial distribution and an identity link to allow interpretations in terms of percentage point increases in overweight/obesity. Since participants contributed varying number of observations, we conducted sensitivity analyses using mixed models with both random intercepts and random intercepts and slopes per participant. Mixed models can offer stronger protection against bias due to missing or available data compared to GEE models [14, 15]. 

To allow for different slopes for each of the time periods, we used spline models to create slope variables. For the primary models we included: baseline age-category, sex, a slope term within each time period, insurance status, and race/ethnicity. We used these primary models to determine slopes for BMI-z scores and the proportion of overweight/obesity for the entire population within each time period, as well as to create point estimates for our outcomes at the start of the study (time 0) and at the end of each of the three study time periods. We also compared the slopes for BMI-z score and the proportion of overweight/obesity between different time periods.

We assessed for effect modification to determine whether there were differences in the slopes for our two outcomes (BMI-z score and overweight/obesity) by (1) age-category, (2) insurance status, and (3) race/ethnicity. We created models with interaction terms between the slope variables within each time period and each potential effect modifier of interest: age-category, insurance status, and race/ethnicity. We assessed statistical significance of each moderator in time period 3 (the COVID-19 pandemic). We then used the models that included the interaction terms to determine point estimates for the subcategories at the start of the study and the end of each study time period. The margins command in STATA was used to determine point estimates for BMI-z score and proportion with overweight/obesity at the start of the study and at the end of each of the three study time periods. All analyses were completed in STATA 17.0 (StataCorp, College Station, TX).

## Results

There were 15,401 children included in the study. 77% received care at primary care sites affiliated with an academic medical system, UCSF Health, and 23% received care at a Federally Qualified Health Center (FQHC) at San Francisco General Hospital. 52% of participants had BMI measurements in two out of three time periods and 48% had BMI measurements from all three study time periods. At baseline, median age was 10 years (IQR: 6–14). 34% were Latino, 26% were White, 20% were Asian, and 9% were Black. 47% were publicly insured (Table 1).

**Table 1:** Demographic characteristics of participants in a study evaluating change in BMI-z scores during the COVID-19 pandemic among Children/Adolescents ages 4–17 in San Francisco, CA

Child Demographics (*n* = 15,*401*)	*N* (%) or median (IQR)
Sex	
Male	7,855 (51%)
Female	7,546 (49%)
Age in years 4–6 year olds 7–9 year olds 10–12 year olds 13–17 year olds	10 (6–14) 3,835 (25%) 3,691 (24%) 2,808 (18%) 5,067 (33%)
Insurance Type	
Public	7,238 (47%)
Private	8,163 (53%)
Race-ethnicity	
Latino	5,236 (34%)
White	4,004 (26%)
Asian	3,080 (20%)
African American	1,386 (9%)
Other (mixed or absent)	1,612 (10%)
American Indian/ Alaskan Indian	49 (0.3%)
Pacific Islander/ Native Hawaiian	34 (0.2%)
Main language spoken at home	
English	12,321 (80%)
Spanish	2,772 (18%)
Other	308 (2%)
Primary care site	
Academic	11,859 (77%)
Federally Qualified Health Center (FQHC)	3,542 (23%)

**Fig. 1:**
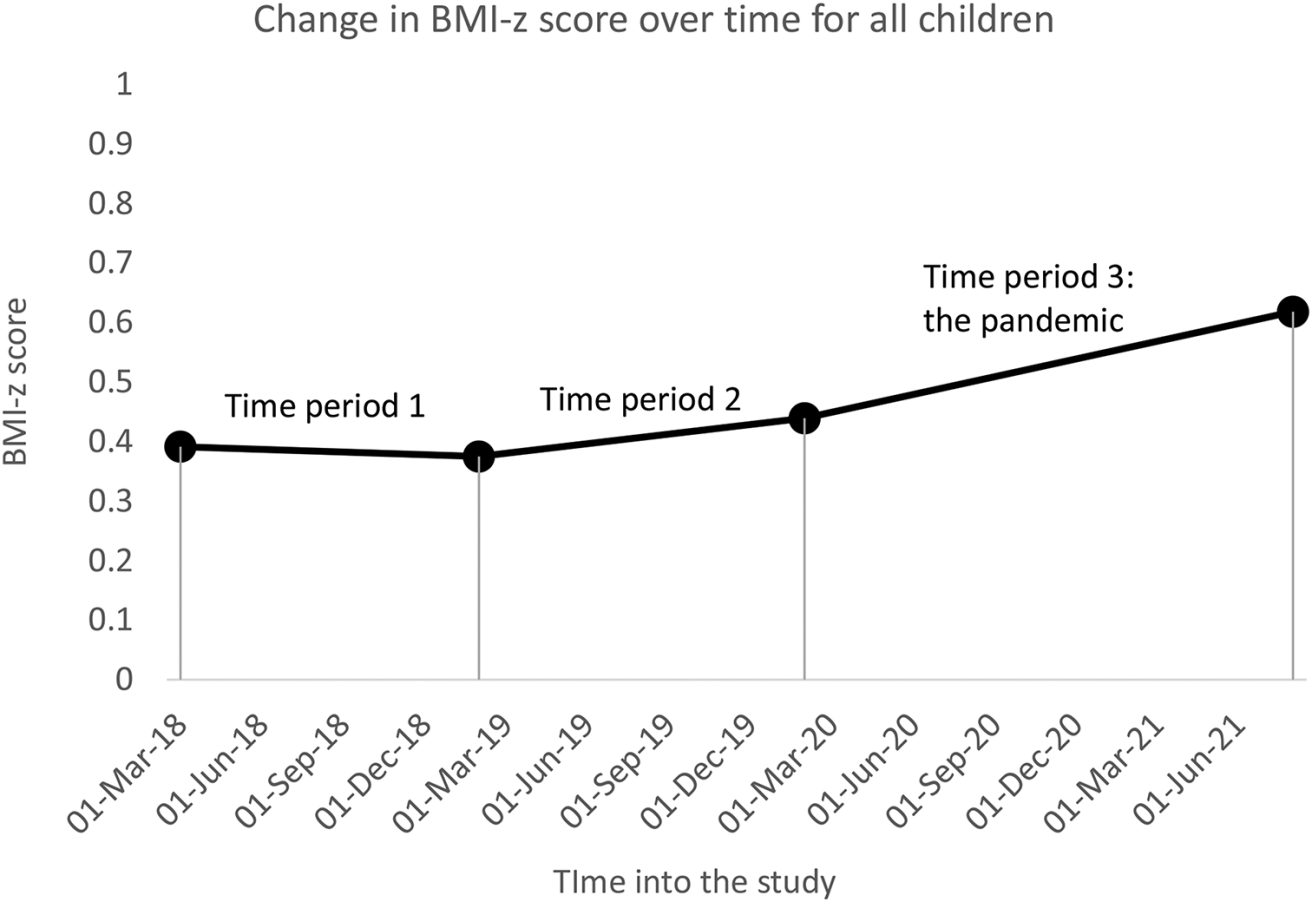
Estimates of change in mean BMI-z score for age and sex over time for total study population. *p* < 0.001 for the comparison of the slope in time period 3 to slope in time period 2

From our primary GEE models, in time period 1, the slope or population level change in BMI-z score per year was − 0.016 (*p* = 0.245, 95% CI: -0.04, 0.01), which was not statistically significant. In time period 2, the year prior to the pandemic, the change in population level BMI-z scores per year was 0.06 (*p* < 0.001, 95% CI: 0.04, 0.09). In time period 3, during the first 18 months of the COVID-19 pandemic, the population level change in BMI-z score per year increased to 0.12 (*p* < 0.001, 95% CI 0.10, 0.13), which was greater than the change in time period 2 (*p* < 0.001). See Fig. 1.

**Fig. 2:**
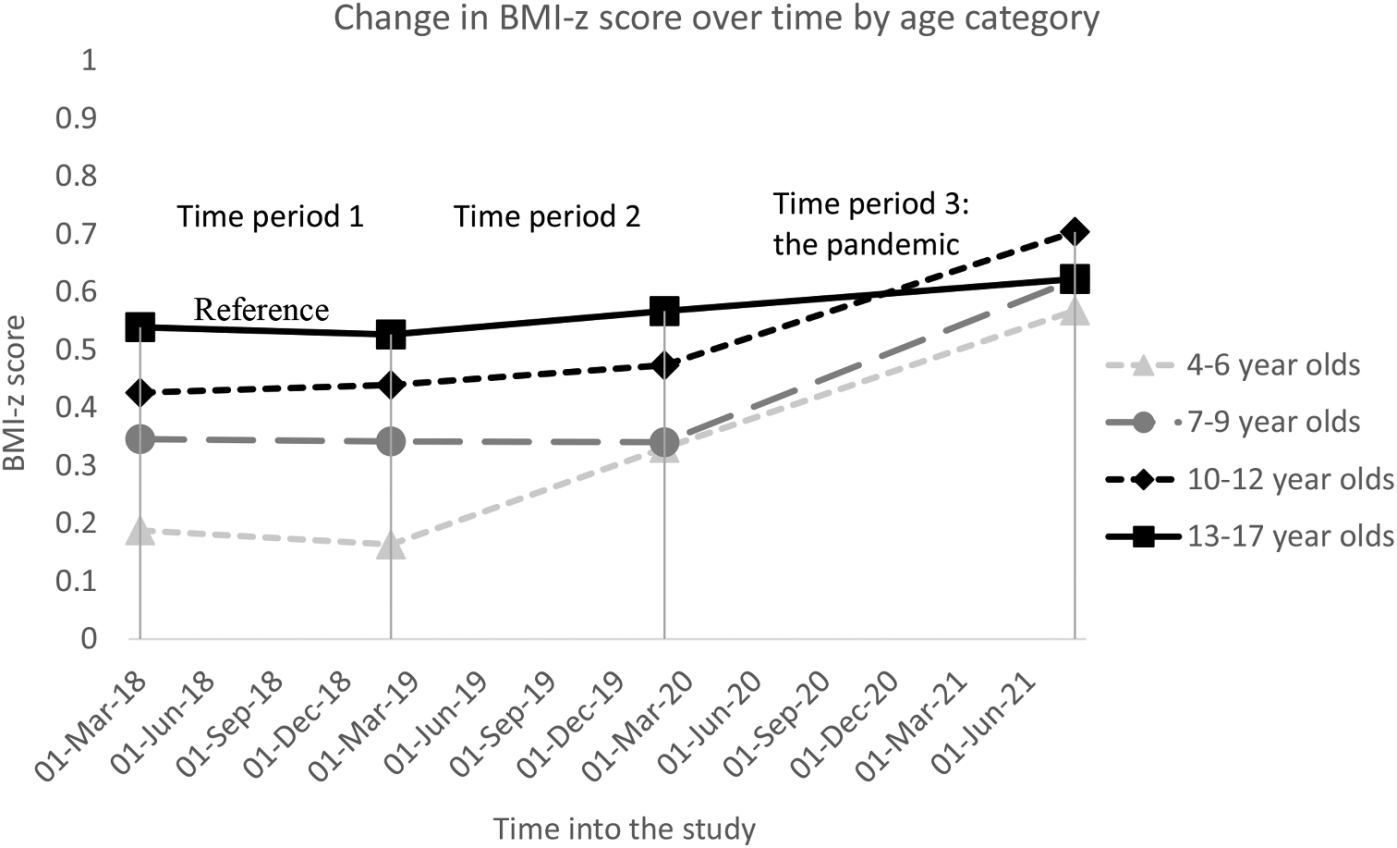
Estimates of change in mean BMI-z score for age and sex over time by age category. In time period 3, *p* < 0.001 for all slope comparisons to the reference group (13–17 year olds)

**Fig. 3:**
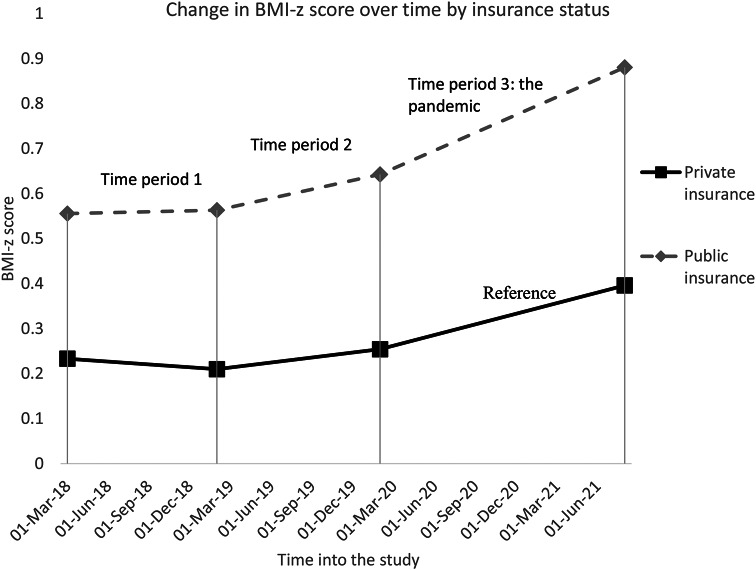
Estimates of change in mean BMI-z score for age and sex over time by insurance status. In time period 3, *p* < 0.001 for slope comparisons to the reference group (private insurance)

**Fig. 4:**
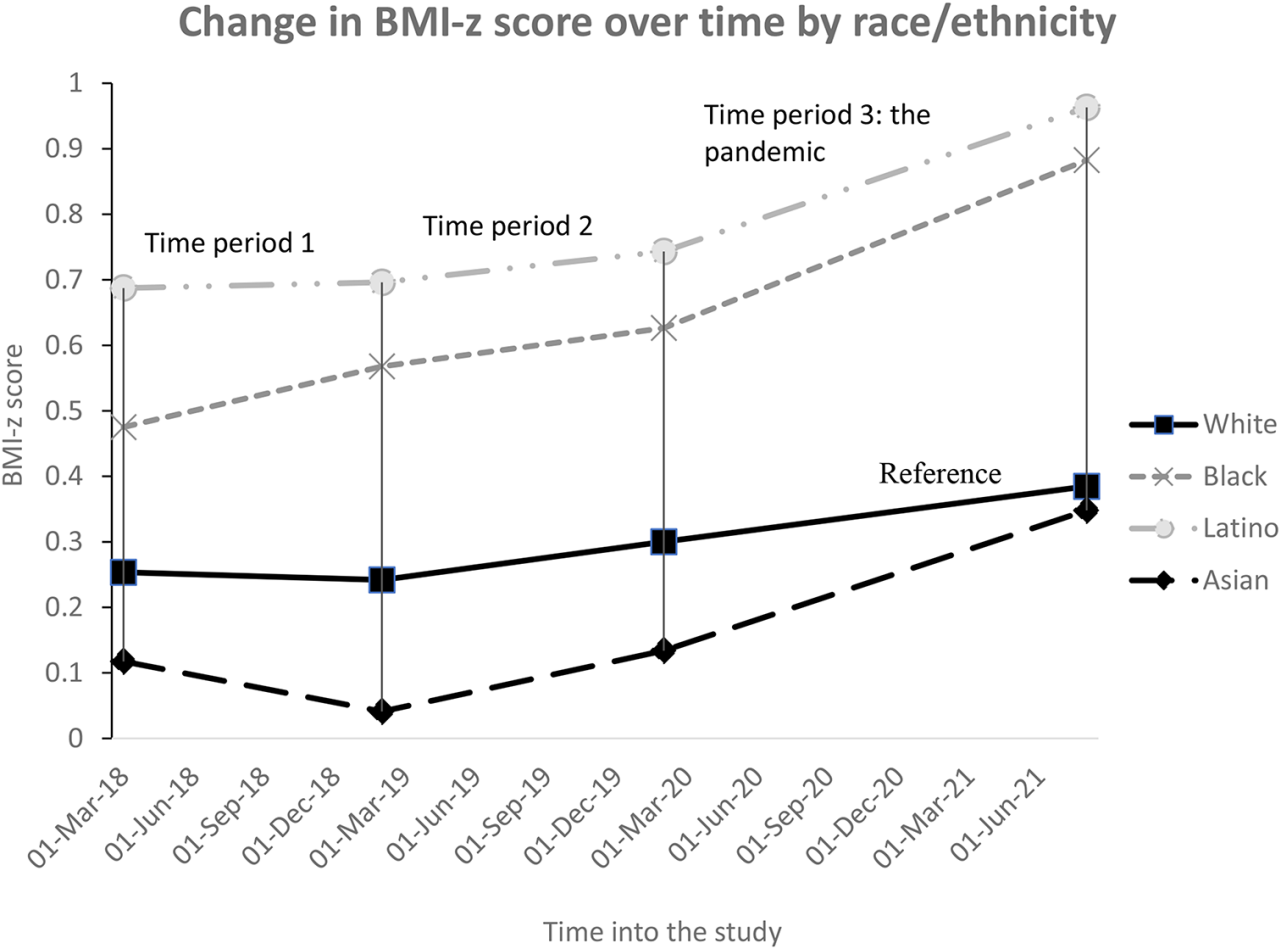
Estimates of change in mean BMI-z score for age and sex over time by race-ethnicity. In time period 3, *p* < 0.001 for all slope comparisons to the reference group (White children)

We found that the following variables were statistically significant effect modifiers of increase in BMI-z score in time period 3 (the pandemic): age-category, insurance status, and race/ethnicity. Children ages 4–6 years, 7–9 years, and 10–12 years old had greater increases in BMI-z score relative to the 13–17 year-olds (*p* < 0.001 for all variables - see Fig. 2). The BMI-z score of publicly insured children increased more rapidly compared to privately insured children (*p* < 0.001 - see Fig. 3) leading to an increase of existing disparities. Black, Latino, and Asian children all had greater increases in BMI-z score during the pandemic compared to White children (*p* < 0.001 for all variables). For Black and Latino children, this contributed to increasing disparities. Asian children, who started off with the lowest BMI-z score, caught up to their White counterparts during the pandemic (Fig. 4).

From our GEE models assessing the proportion of children with overweight/obesity, during time period 1, the increase of children with overweight/obesity was nonsignificant. In time period 2, the proportion of children with overweight/obesity increased by 1.38% points per year (*p* = 0.012, 95% CI: 0.03, 2.46). In time period 3, the COVID-19 pandemic, the proportion of children with overweight/obesity, increased by 4.89% points per year (*p* < 0.001, 95% CI: 4.11, 5.67).

We found that the following variables were statistically significant effect modifiers of the change in the proportion of children with overweight/obesity in time period 3 (the COVID-19 pandemic): age-category and insurance status. Children ages 4–6 years (*p* = 0.022), 7–9 years (*p* < 0.001), and 10–12 years old (*p* = 0.001) had greater increases in proportion of overweight/obesity compared to children 13–17 years of age. Children with public insurance had greater increases in proportion of overweight/obesity compared to children with private insurance (*p* < 0.001). We did not find significant results for moderation by race/ethnicity. Table 2 shows changes over time in proportion of children with overweight/obesity overall, by age-category, insurance status, and by race/ethnicity. As with BMI-z score, we observed widening disparities in the prevalence of overweight/obesity. At the end of time period 2 (prior to the pandemic), the difference in the prevalence of overweight/obesity between children with private and public insurance was 12.8% points (21.5% vs. 34.3%). At the end of time period 3, 18-months into the pandemic, this disparity had widened to 20% points (26.6% vs. 46.6%). The sensitivity analyses using the mixed models gave virtually identical results to the GEE analyses, except for the p-value for the youngest children, which became non-significant in the overweight/obesity model (data not shown). This provides some assurance the missing data did not bias the results.

**Table 2:** Model estimates of the percent of children with Overweight/Obesity in each study Time Period by Age Category, Insurance Status, and Race/Ethnicity

	Start of the study: March 1, 2018	End of time period 1: February 28, 2019	End of time period 2: February 28, 2020	End of time period 3 (the COVID-19 pandemic): August 31, 2021
Percent of all children with overweight/obesity (%)	25.0	26.1	28.2	36.2
Age category:				
4–6 year olds * 7–9 year olds ** 10–12 year olds ** 13–17 year olds= (reference group)	20.8 17.5 25.8 35.1	15.8 20.7 28.4 35.9	18.5 21.9 33.8 36.4	25.5 36.9 43.3 40.7
Insurance Category:				
Public insurance** Private insurance= (reference group)	31.1 19.8	32.6 18.9	34.3 21.5	46.6 26.6
Race/ethnicity:				
Black Latino Asian White= (reference group)	30.9 34.9 17.7 18.2	31.1 34.5 17.1 19.8	34.3 38.3 18.3 20.3	43.2 48.0 27.9 26.4

* *p* < 0.05 for effect modification of the increase in overweight-obesity relative to the reference group during time period 3

* *p* ≤ 0.001 for effect modification of the increase in overweight-obesity relative to the reference group during time period 3

## Discussion

Our study of children in San Francisco, CA found that both mean BMI-z score and the proportion of children with overweight/obesity increased significantly during the first 18-months of the COVID-19 pandemic (time period 3: when public schools were closed) compared to prior secular changes. The BMI-z score increase seen in time period 2 is most likely attributable to the aging of the cohort given that overweight/obesity increases with age and CDC growth curves are primarily based on growth of children from time periods that preceded the obesity epidemic. The mean BMI-z score for all children increased twice as rapidly during the pandemic compared to the year prior (0.12 per year vs. 0.06 per year prior to the pandemic). The estimated proportion of children overall with overweight/obesity increased from 28.2% prior to the pandemic to 36.2% after 18 months into the pandemic. Our study revealed greater increases in BMI-z scores among younger, publicly insured, Latino, Black, and Asian children relative to children who were White, older, and had private insurance during the pandemic time-period. This led to widening health disparities as Black, Latino, and publicly insured children started off the study with a significantly higher mean BMI-z score and subsequently had greater increases during the pandemic. Notably, the increase in BMI-z scores among Latino children during the pandemic was 2.8 times greater than that seen in White Children; for Black children the increase was 3.2 times greater than that seen in White children during the pandemic.

Our findings are an important addition to the emerging literature on children’s weight trajectories during the COVID-19 pandemic, which have consistently shown increases in the rate of weight gain and elevated BMI in the first year of the pandemic [1–8]. More recent retrospective cohort studies examining the impacts over a 18–30 month period show a more complex pattern. A recent study in Indianapolis found that higher rates of severe obesity persisted over the 18-month follow-up period and that rates of overweight/obesity persisted for children ages 5–11 years [11]. This is consistent with our findings that elementary aged children were most impacted by the pandemic. A recent study in Philadelphia found that the prevalence of obesity increased early on in the pandemic among children and then returned to pre-pandemic levels by December 2022, though sociodemographic disparities persisted [10]. It is not clear whether San Francisco and other locations will follow this long-term pattern evident in Philadelphia or whether the increases in the rates of overweight/obesity will continue to persist. It is possible that in San Francisco and other jurisdictions where pandemic restrictions were more severe and of longer duration, that changes in overweight/obesity will persist for longer periods. This is an important area for further research.

The emerging literature has also shed light on how ethnic and racial disparities in children/adolescents’ health that existed prior to the onset of COVID-19 have been exacerbated by the pandemic [1, 3, 4, 9]. A large national retrospective cohort study as well as other local studies found that children who were Black or Latino had the highest BMI prior to the pandemic, and also experienced the largest increase in weight gain during the pandemic [1, 3, 4, 9]. In our study, we used insurance status as a proxy for family level socio-economic status [16, 17] and found children with public insurance had greater increases in both BMI-z score and overweight/obesity compared to those with private insurance. This suggests that families with fewer resources may have had less capacity for buffering the effects of the pandemic on their children’s weight trajectories. Our finding that BMI-z score and overweight/obesity increased more in younger children versus adolescents also contributes to this emerging literature on the impacts of COVID on childhood obesity. However, the explanations for these age differences are not fully understood and could benefit from further research.

Families in San Francisco, CA were eligible for many expanded public benefits offered at the federal, state and local level during the pandemic, including increases in Cal Fresh (California’s supplemental nutrition assistance program), a federal child tax credit, and the pandemic electronic benefit transfer program (a federal program in the United States that provided funds to families with children for food purchases) [18–21]. It is important to note, however, that families with mixed documentation status were excluded from some federal COVID relief efforts and had more limited access to other expanded public benefits. Our study suggests that expanded public benefits did not compensate for the hardships created through the pandemic. With decreased access to school meals and widespread unemployment in the service sector, many families in San Francisco experienced worsening household food insecurity, particularly families with young children [5, 18, 19]. Studies also suggest that families relied more on ultra-processed shelf-stable foods, a known risk factor for obesity [22–24]. School closures and restrictions on the use of public spaces may also have limited physical activity among San Francisco’s school age children. Playgrounds in San Francisco were closed for the first six months of the pandemic, and after they opened, there was signage that limited play to 30 min. In addition, families in communities that were most impacted by COVID may have been more hesitant to allow children to play in communal spaces, even after it was allowed. Public policies to compensate for the effects of the pandemic were likely not of sufficient magnitude or targeted adequately to high-risk children to prevent the increases in overweight/obesity found in this study.

Our study has multiple strengths. This study captures a long time period of 18-months into the pandemic that defined school closure policies in this community. BMI data were based on measured heights and weights taken from medical records from in-person medical visits, having an advantage over many studies that relied on self-reported weights. Finally, our study sample is highly diverse, reflecting the diversity of the San Francisco Bay Area, and promoting greater understanding of pandemic impact on multiple diverse groups of children. Our study has several important limitations. We were restricted to children/adolescents who accessed medical care in San Francisco during the pandemic, thus limiting the generalizability of the study findings. Because we utilized medical record data, if availability of data were related to trends in BMI (e.g., participants whose BMI is increasing are measured more frequently) it may cause bias. We also lacked access to data on household food insecurity or other social drivers of health that could be mediating the relationship between pandemic policies and increasing weight gain/obesity.

## Conclusions

In a diverse group of children receiving primary care in San Francisco, we found significant increases in both BMI-z score and the prevalence of overweight/obesity during the first 18 months of the COVID-19 pandemic (the duration of school closures for most children) compared to the pre-pandemic period among all populations examined. We also found widening disparities in BMI among children with public insurance and children of color. If these increases in BMI status persist over time, they are likely to increase these children’s risk for numerous adverse cardiometabolic outcomes. These results highlight the need for more effective public policies for obesity prevention and treatment, through support for physical activity programs and access to healthy foods. The current imperative is to ensure that resources are directed to groups with high rates of overweight/obesity.

In addition, given the consequences of excess weight gain and new or worsening obesity on future cardiometabolic risk, policy makers must consider the risk of increasing weight gain when considering future pandemic policies including school and recreational program closure and efforts to alleviate economic distress and improve access to healthy foods.

## Data Availability

The datasets used and/or analyzed during the current study are available from the corresponding author on reasonable request.
